# Narrative review of intravenous NAD^+^ and NAD^+^ precursors in wellness and translational medicine

**DOI:** 10.3389/fragi.2026.1887175

**Published:** 2026-09-10

**Authors:** Abdulrahman Alangari, Jamal Arif, Fahd Al Qureshah, Fahad Alkhodairy

**Affiliations:** 1 Department of Research & Development, Evercare, Riyadh, Saudi Arabia; 2 Department of Gastroenterology and Hepatology, Prince Sultan Military Medical City, Riyadh, Saudi Arabia; 3 College of Medicine, Shaqra University, Shaqra, Saudi Arabia; 4 Bioscience Program, King Abdullah University of Science and Technology (KAUST), Thuwal, Saudi Arabia

**Keywords:** clinical wellness settings, cognitive function, intravenous, metabolic health, NAD^+^ therapy, nicotinamide mononucleotide, oral

## Abstract

**Background:**

Nicotinamide adenine dinucleotide (NAD^+^) is a major coenzyme critically involved in cellular metabolism, mitochondrial function, DNA repair, and stress-response signaling. Age-associated decline in NAD^+^ levels has generated interest in NAD^+^-augmenting strategies; however, recent large-scale human data indicate that whole-blood NAD^+^ concentrations do not decline with healthy aging *per se*. Despite increasing commercial use of IV NAD^+^ therapy in wellness settings, the clinical evidence supporting its efficacy and long-term safety remains limited.

**Methods:**

Literature published between January 2018 and March 2026 was identified *via* PubMed and Google Scholar searches. Search terms were applied symmetrically across all target compounds and administration routes. Studies were included if they reported human clinical data on IV NAD^+^ or IV NAD^+^ precursor administration, or if they provided directly relevant mechanistic or safety evidence. Mechanistic and foundational studies published before 2018 were selectively incorporated *via* citation tracking of key included articles.

**Results:**

Available human evidence is sparse and consists primarily of small uncontrolled studies, observational investigations, and isolated case reports. Short-term IV NAD^+^ or NMN administration has been associated with transient increases in circulating NAD^+^ levels and changes in selected biomarkers related to oxidative stress, inflammation, and cellular metabolism. Limited exploratory reports have also described self-reported improvements in sleep-related outcomes and neurologic symptoms. However, the clinical significance of these findings remains uncertain due to small sample sizes, lack of placebo controls, heterogeneous methodologies, and short follow-up durations. Reported adverse effects include nausea, cramping, flushing, and chest discomfort, while long-term safety data are lacking.

**Conclusion:**

Current evidence regarding IV NAD^+^ therapy remains preliminary and insufficient to support routine clinical or wellness use. Although mechanistic and translational studies provide biological rationale for NAD^+^ augmentation, robust conclusions regarding efficacy, durability of benefit, optimal dosing, and long-term safety cannot currently be established. Larger randomized controlled trials with standardized protocols and clinically meaningful endpoints are required before IV NAD^+^ therapy can be considered evidence-based clinical practice.

## 1 Introduction

Nicotinamide adenine dinucleotide (NAD^+^) is one of the most recognized fundamental coenzymes in physiology, involved in cellular energy metabolism, redox reactions, DNA repair, epigenetic regulation, and cellular signaling pathways (Xie et al., 2020). NAD^+^ is a crucial component of mitochondrial function and cellular homeostasis, involved in more than 500 enzymatic reactions and being the indispensable substrate for three key enzyme families: sirtuins, poly(ADP-ribose) polymerases, and cyclic ADP-ribose synthases (CD38/CD157) (Verdin, 2015). NAD^+^ progressively declines over time, making it central to age-related metabolic dysfunction, mitochondrial dysfunction, senescence of the cell, as well as increased susceptibility to chronic diseases (Yoshino et al., 2018). This widely held premise deserves critical scrutiny; however, Trętowicz et al. (2026), in a large-scale cross-sectional cohort study, demonstrated that whole-blood NAD^+^ concentrations were not independently associated with chronological age once disease burden and comorbidity were accounted for. Janssens et al. (2022) similarly argued that the clinical relevance of NAD^+^ decline should be evaluated in the context of metabolic dysfunction rather than aging *per se*. These findings carry practical implications for patient selection in IV NAD^+^ trials: if age alone does not reliably predict NAD^+^ deficiency, trials may benefit from screening participants by whole-blood NAD^+^ status rather than by age bracket alone, to enrich for individuals most likely to demonstrate measurable responses to repletion therapy.

The realization that NAD^+^ depletion is a property of aging/age-associated pathologies has led to intensive investigation of therapeutic avenues to restore NAD^+^ homeostasis (Rajman et al., 2018). NAD^+^ can be synthesized across multiple biosynthetic pathways, which include the *de novo* pathway of tryptophan, the Preiss-Handler pathway of nicotinic acid, and the salvage pathway from nicotinamide (NAM), nicotinamide riboside (NR), and nicotinamide mononucleotide (NMN) (Yoshino et al., 2018). Within this cascade, a salvage pathway mediated by nicotinamide phosphoribosyltransferase (NAMPT) has been found to dominate mammalian tissues and should be the focus of NAD^+^ augmentation strategies (Ratia et al., 2023).

Supplementation strategies aimed at increasing NAD^+^ availability have attracted growing scientific interest because age-related decline in NAD^+^ has been associated with mitochondrial dysfunction, impaired metabolic regulation, and altered cellular stress responses. Preclinical studies suggest that NAD^+^ augmentation may influence pathways involved in energy metabolism, oxidative stress regulation, DNA repair, and inflammatory signaling (Kane et al., 2024-preprint). Early human studies evaluating oral NAD^+^ precursors such as NR and NMN have reported changes in selected metabolic and inflammatory biomarkers, although findings remain inconsistent and clinical significance is uncertain (Gan, 2022; Liao et al., 2024). These observations have encouraged investigators to explore alternative delivery methods, including intravenous (IV) administration, which theoretically bypasses gastrointestinal absorption and first-pass metabolism (Benjamin and Crews, 2024; Reyna et al., 2026). Although IV administration may achieve rapid systemic exposure, direct evidence demonstrating superiority over oral supplementation is currently lacking (Kimura et al., 2022). Furthermore, many IV infusion protocols described in commercial settings have not undergone formal clinical validation, and substantial variation exists in dosing strategies, infusion duration, and accompanying supplements.

Several commercial concerns have emerged regarding the expansion of IV NAD^+^ therapy in wellness settings before standardized protocols, long-term safety data, or guideline-supported indications are established. Much of the currently available literature consists of small observational studies, uncontrolled investigations, mechanistic reports, and translational hypotheses rather than adequately powered randomized clinical trials (Gindri et al., 2024; Sohouli et al., 2024). In addition, important uncertainties remain regarding comparative effectiveness between IV and oral administration, durability of reported effects, optimal dosing schedules, patient selection, and long-term safety outcomes (Benjamin and Crews, 2024).

Thus, this narrative review critically examines the current evidence relating to IV NAD^+^ therapy and related precursor administration in human subjects, the quality and certainty of available data, and the identification of key gaps requiring prospective investigation. The review is intended to provide a structured, evidence-graded synthesis for clinicians and researchers working in this area.

## 2 Methods

### 2.1 Search strategy and selection of studies

This manuscript was conducted as a structured narrative review intended to summarize and critically evaluate the current clinical and translational literature relating to IV NAD^+^ therapy and its precursors. Given the limited availability of RCTs and the substantial heterogeneity of the existing literature, a formal systematic review and meta-analysis approach was not considered appropriate.

Literature searches were performed using PubMed and Google Scholar for studies published between January 2018 and March 2026. This date range was applied specifically to clinical and translational searches. Foundational mechanistic studies published before 2018 were selectively incorporated *via* citation tracking of included articles when essential to providing biological context for NAD^+^ biology and the rationale for IV administration. Search terms included combinations of “NAD^+^ intravenous,” “NAD^+^ infusion,” “intravenous nicotinamide mononucleotide,” “NMN IV,” “nicotinamide riboside,” “NAD^+^ metabolism,” “sirtuins,” “mitochondrial function,” “healthy aging,” and related terms. Reference lists of relevant articles were also screened to identify additional studies.

Studies were included if they met the following criteria: (1) human clinical studies, observational investigations, or case reports evaluating IV or oral NAD^+^, NMN, NAM, or NR administration; (2) mechanistic or translational studies providing a biological context relevant to IV NAD^+^ administration; (3) preclinical studies included only where they provided a mechanistic or translational context. Studies focused exclusively on unrelated disease-specific interventions without relevance to NAD^+^ biology or IV administration were excluded. Studies were screened first by title and abstract, and then by full-text review.

### 2.2 Quality and certainty of evidence

The overall quality of evidence relating to IV NAD^+^ therapy remains low. Most available human data derive from small, uncontrolled studies, observational reports, and isolated case reports with limited follow-up durations. Randomized placebo-controlled trials specifically evaluating IV NAD^+^ administration are currently lacking. As a result, substantial risks of selection bias, placebo effects, confounding, and overinterpretation of biomarker findings remain.

Because of substantial differences in study design, population characteristics, interventions, and reported outcomes, findings were synthesized qualitatively rather than quantitatively. Particular attention was given to study design, sample size, the presence or absence of control groups, the duration of follow-up, and the clinical applicability of the reported findings. Many reported outcomes are exploratory surrogate biomarkers rather than validated clinical endpoints. Improvements in laboratory or molecular markers should therefore not be interpreted as confirmation of clinically meaningful anti-aging, cognitive, neurologic, or metabolic benefit. Further, evidence derived from mechanistic studies, observational investigations, and case reports was interpreted cautiously and was not considered equivalent to evidence from RCTs.

Several publications included in the literature are also susceptible to commercial or publication bias, particularly within wellness-oriented settings where IV NAD^+^ therapies are increasingly marketed despite limited supporting evidence. Consequently, the current body of literature should be regarded as hypothesis-generating rather than practice-changing evidence.

## 3 Results

### 3.1 Mechanisms of action for IV NAD^+^ therapy

**FIGURE 1:**
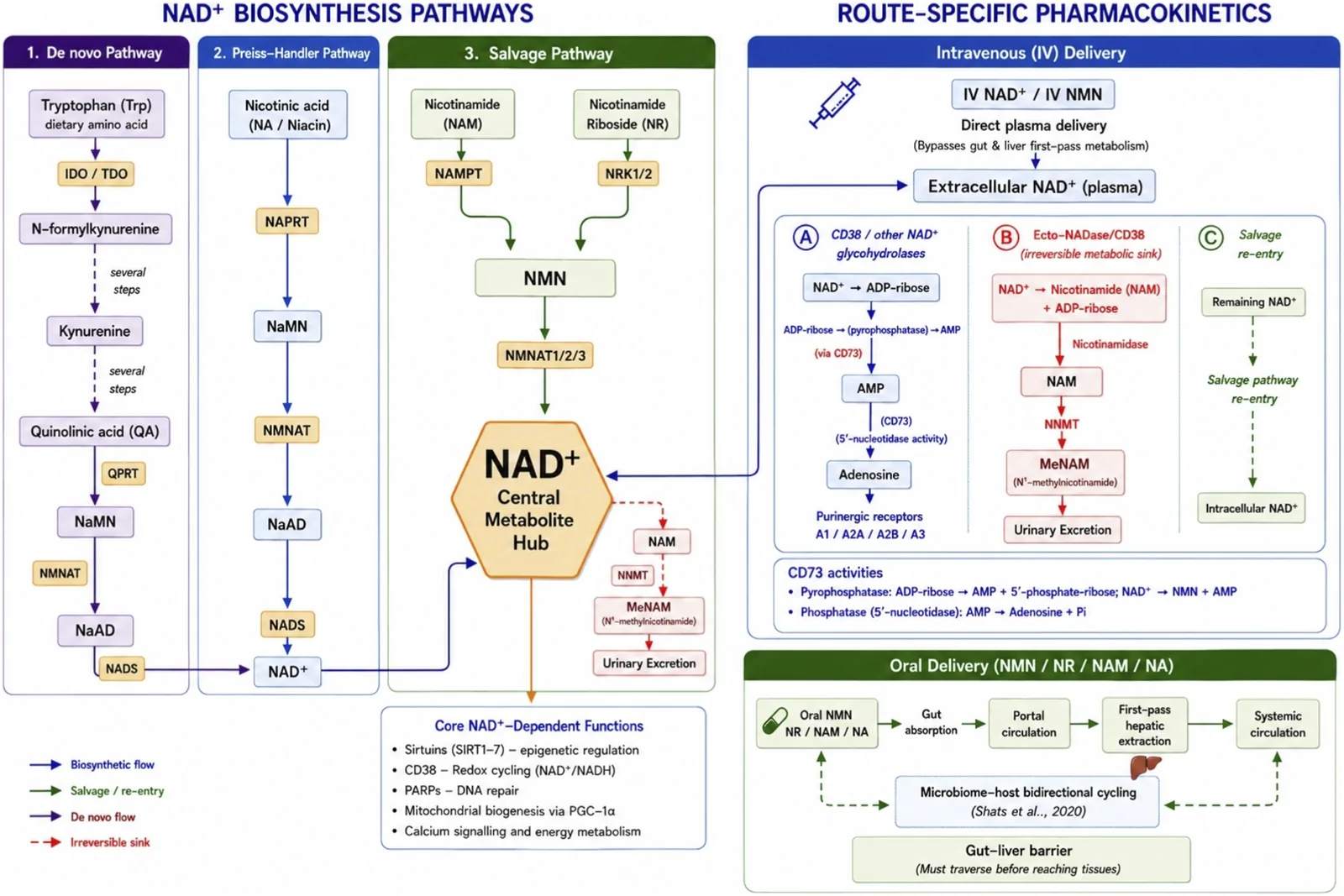
NAD^+^ Biosynthesis Pathways and Route-Specific Pharmacokinetics. Schematic overview of the three major pathways involved in NAD^+^ biosynthesis: the *de novo* pathway from tryptophan, the Preiss–Handler pathway from nicotinic acid (NA), and the salvage pathway involving nicotinamide (NAM) and nicotinamide riboside (NR). In the *de novo* and Preiss–Handler pathways, NaMN is converted to NaAD and subsequently to NAD^+^ by NADS, whereas the NAM and NR salvage routes converge at NMN, which is converted to NAD^+^ by NMNAT1/2/3. NAMPT is highlighted as the key rate-limiting enzyme in the NAM salvage pathway. The figure also compares the route-specific pharmacokinetic features of IV and oral NAD^+^ precursors. IV NAD^+^ or IV NMN enters the systemic circulation directly, bypassing gastrointestinal absorption and first-pass hepatic metabolism of the administered compound. Extracellular NAD^+^ can undergo metabolism by NAD^+^-consuming enzymes, including CD38, generating NAM and ADP-ribose. ADP-ribose can undergo further extracellular metabolism to AMP, which CD73 subsequently dephosphorylates to form adenosine. The figure also depicts extracellular NAD^+^ pyrophosphatase activity yielding NMN and AMP; this reaction should be distinguished from the AMP phosphatase activity of CD73. These extracellular metabolites may contribute to purinergic signalling, although their contribution to the clinical effects of IV NAD^+^ in humans remains uncertain. NAM can re-enter NAD^+^ biosynthesis through salvage or undergo irreversible methylation by nicotinamide N-methyltransferase (NNMT) to form N^1^-methylnicotinamide (MeNAM), which can be excreted in urine. Oral NAD^+^ precursors undergo gastrointestinal absorption, portal circulation, and first-pass hepatic extraction before reaching the systemic circulation, with additional modulation by microbiome–host metabolism. NAD^+^ generated through these pathways forms a central metabolic pool that serves as a substrate for sirtuins, PARPs, and CD38-associated metabolism and contributes to mitochondrial and cellular energy-related functions. The figure also illustrates the potential re-entry of NAD^+^ breakdown products into the salvage pathway. Abbreviations: IDO, indoleamine 2,3-dioxygenase; TDO, tryptophan 2,3-dioxygenase; QPRT, quinolinate phosphoribosyltransferase; NAPRT, nicotinic acid phosphoribosyltransferase; NAMPT, nicotinamide phosphoribosyltransferase; NMNAT, nicotinamide mononucleotide adenylyltransferase; NRK, nicotinamide riboside kinase; NADS, NAD synthetase; NNMT, nicotinamide N-methyltransferase; CD38, cyclic ADP-ribose hydrolase/glycohydrolase; CD73, ecto-5′-nucleotidase; MeNAM, N^1^-methylnicotinamide; NaMN, nicotinic acid mononucleotide; NaAD, nicotinic acid adenine dinucleotide; NAD^+^, nicotinamide adenine dinucleotide; NMN, nicotinamide mononucleotide; NR, nicotinamide riboside; NAM, nicotinamide.

IV administration places NAD^+^ directly into the systemic circulation, where it may undergo extracellular metabolism before cellular uptake. It remains uncertain how much intact NAD^+^ enters cells compared with the contribution of metabolites such as NAM and NMN to intracellular NAD^+^ replenishment. This distinction is important when interpreting the pharmacological effects of IV NAD^+^. The pharmacokinetic distinctions between IV and oral routes of NAD^+^ precursor delivery, and the principal enzymatic steps governing NAD^+^ biosynthesis and extracellular metabolism, are summarised schematically in Figure 1.

An important pharmacological consideration for IV NAD^+^ administration concerns the metabolic fate of exogenously delivered NAD^+^ in the systemic circulation. Following IV infusion, NAD^+^ is subjected to extracellular hydrolysis by ectonucleotidases, most notably CD38, which cleaves NAD^+^ into NAM and ADP-ribose and other downstream metabolites before cellular uptake can occur (Camacho-Pereira et al., 2016; Benzi et al., 2022; Ratia et al., 2023). As a consequence, it remains uncertain whether circulating NAD^+^ is internalized intact or whether the observed biological effects are primarily mediated through the cellular uptake of degradation products, particularly NAM and NMN, which are subsequently re-incorporated into intracellular NAD^+^ *via* the salvage pathway (Trammell et al., 2016; Yoshino et al., 2018). This distinction is important when interpreting the pharmacokinetic and pharmacodynamic effects of IV NAD^+^. Rigorous human pharmacokinetic studies are needed to define the plasma kinetics of intact NAD^+^, its tissue distribution, and the relative contributions of intact NAD^+^ *versus* downstream metabolites to observed biological effects (Rajman et al., 2018; Yoshino et al., 2018; Grant et al., 2019).

The extracellular metabolism of NAD^+^ should be described more carefully because several enzymatic activities have been assigned to CD73-related pathways. Extracellular NAD^+^ can undergo pyrophosphatase activity that yields NMN and AMP, while CD73 (ecto-5′-nucleotidase) is best established as an AMP phosphatase that generates adenosine. Wilk et al. (2020) found that recombinant human CD73 did not appreciably process NAD^+^ and only poorly processed NMN, whereas the bacterial NAD-processing orthologue examined in that study could convert NAD^+^ to NMN and AMP but did not convert NMN to NR. Thus, the extracellular conversion of NAD^+^ to NMN/AMP and the subsequent formation of adenosine should not be attributed indiscriminately to human CD73.

Following extracellular NAD^+^ cleavage by NAD^+^ glycohydrolases (such as CD38), NAM and ADP-ribose can be generated. ADP-ribose may undergo further extracellular metabolism to AMP, while AMP is dephosphorylated by CD73 to adenosine. In parallel, extracellular NAD^+^ pyrophosphatase activity can generate NMN and AMP. These pathways provide a plausible route for adenosine formation after IV NAD^+^ administration, but the relative contribution of each enzyme and the extent to which these reactions occur *in vivo* in humans remain uncertain (Grant et al., 2019; Wilk et al., 2020).

A further pharmacological constraint relevant to IV NAD^+^ is the irreversible methylation of NAM by nicotinamide N-methyltransferase (NNMT), which converts NAM to N^1^-methylnicotinamide (MeNam) in a reaction that cannot re-enter the NAD^+^ biosynthetic salvage pathway (Wilk et al., 2020). The partitioning of released nicotinamide between NNMT-mediated catabolism and NAMPT-mediated salvage is cell-type-dependent, and the fraction irreversibly lost to NNMT activity represents a direct constraint on the net NAD^+^ yield per administered dose of IV NAD^+^. This ‘one-hit’ loss is not detectable from whole-blood NAD^+^ measurements alone and may be quantified indirectly through urinary MeNam excretion as a supplementary endpoint in future pharmacokinetic trials.

The increased intracellular NAD^+^ activates the three major enzyme families regulating the therapeutic effect of NAD^+^ supplementation. Sirtuins comprise a family of seven NAD^+^ -dependent deacetylases (SIRT1-7) involved in a range of cellular processes such as energy metabolism, mitochondrial biogenesis, DNA repair, inflammation, and stress resistance (Verdin, 2015). The largest known family member, SIRT1, deacetylates important transcription factors including FOXO1, PGC-1α, and p53, enhancing mitochondrial functionality, boosting antioxidant defenses, and fostering cellular survival pathways (Braidy, 2018). One of the paradigmatic human studies showed that 6 days of IV NAD^+^ (1,000 mg daily) administered in elderly adults significantly upregulated SIRT1 mRNA expression and activity in peripheral blood mononuclear cells, and increased FOXO1 expression and reduced acetylated p53 (Braidy, 2018). Molecular changes such as these reflect entry into signaling pathways associated with longevity that might underlie anti-aging actions.

Poly(ADP-ribose) polymerases, another key NAD^+^-demanding enzyme family involved in the detection and repair of DNA damage, maintain genomic stability and regulation of cell death pathways (Xie et al., 2020). PARP activation in response to DNA damage requires a substantial amount of NAD^+^, and prolonged overactivation of PARP during aging and oxidative stress can lead to depletion of NAD^+^ stores in cells (Verdin, 2015), establishing a vicious cycle of metabolic dysfunction. The IV therapy can restore NAD^+^ and support efficient DNA repair while minimizing excessive depletion of NAD^+^, which would otherwise compromise the energy metabolism of the cell.

CD38, a multifunctional enzyme with NAD^+^ glycohydrolase activity, represents the primary NAD^+^ consumer in many tissues and a key driver of age-related NAD^+^ decline (Benzi et al., 2022). Increasing expression of CD38 with age and inflammation can accelerate the degradation of NAD^+^ and contribute to metabolic dysfunction (Benzi et al., 2022; Zhao et al., 2025). Preclinical studies demonstrate that CD38 inhibition or deletion increases tissue NAD^+^ levels and improves metabolic health during thermogenesis and aging (Benzi et al., 2022). IV NAD^+^ treatment will probably not necessarily directly inhibit CD38 but may alleviate the deficit of CD38-mediated NAD^+^ ingestion *via* the provision of exogenous NAD^+^ substrate (Camacho-Pereira et al., 2016).

At the cellular level, increased NAD^+^ provides several mechanisms to promote mitochondrial function. Increased NAD^+^ availability supports the electron transport chain, allowing an increase in the efficiency of ATP production and a reduction in reactive oxygen species generation (Crunkhorn, 2018). SIRT1 activation promotes mitochondrial biogenesis *via* deacetylation of PGC-1α, leading to an increase in mitochondrial mass and oxidative capacity (Braidy, 2018). Preclinical investigations of differentiated PC12 cells show that NAD^+^ treatment affects the glutathione/oxidized glutathione (GSH/GSSG) ratio by a SIRT2-, ERK-, and Nrf2-dependent upregulation of gamma-glutamyl cysteine ligase, the rate-limiting enzyme for glutathione biosynthesis (Zhang et al., 2018). This increase in antioxidant capacity is a key mechanism by which NAD^+^ therapy might protect against oxidative stress and cellular damage.

The salvage pathway enzyme NAMPT is the rate-limiting step in NAD^+^ biosynthesis from NAM, and its activity declines with aging (Ratia et al., 2023). Recent mechanistic studies have shown that positive allosteric regulators of NAMPT strongly influence enzyme activity and cellular NAD^+^ levels by relieving feedback inhibition and enhancing enzyme stability (Ratia et al., 2023). While IV NAD^+^ bypasses the NAMPT bottleneck by directly supplying the NAD^+^ substrate, understanding NAMPT regulation remains key to developing optimized combination strategies that improve both NAD^+^ production and endogenous biosynthesis (Rajman et al., 2018).

An important pharmacokinetic consideration unique to oral NAD^+^ precursor administration is the potential for the intestinal microbiota to compete with host tissues for available NMN and NR substrates. Shats et al. (2020) demonstrated in murine models that commensal bacteria actively consume NMN and NR through a deamidated biosynthetic pathway, substantially reducing the fraction of orally ingested precursor that reaches systemic circulation. Subsequently, Chellappa et al. (2022) characterised bidirectional cycling of NAD^+^ precursors between host tissues and the gut microbiome in humans, further establishing that the gut microbiome is an active participant in, rather than a passive bystander to, NAD^+^ precursor metabolism. IV administration of NAD^+^ entirely bypasses the gastrointestinal tract and therefore avoids this microbial competition, ensuring complete systemic bioavailability of the administered dose; however, the comparative clinical significance of this theoretical advantage over oral precursors has not been directly studied.

Interpretation of circulating NAD^+^ measurements also requires attention to pre-analytical conditions. Whole-blood NAD^+^ is sensitive to the timing of blood collection and to sample handling before analysis. Recent human work has shown that storage temperature, freezing and freeze–thaw handling can materially alter measured NAD^+^ concentrations, while differences in sampling conditions may add biological and technical variability (Trętowicz et al., 2026). Timing may also matter biologically because NAD^+^ salvage is influenced by the diurnal regulation of NAMPT and related pathways. Fasting status is another potential source of variation and should be standardized when comparing intervention studies. The Grant et al. (2019) study illustrates the value of this approach by using an overnight fast and immediate processing and storage of samples at −80 °C. Accordingly, future IV NAD^+^ studies should report collection time, fasting status, processing interval, storage temperature, freeze–thaw history, and the analytical method in sufficient detail to permit meaningful comparison across studies.

IV administration bypasses gastrointestinal absorption and first-pass metabolism and therefore places the administered dose directly into the systemic circulation. This can reasonably be described as complete bioavailability with respect to entry into the circulation, but it does not demonstrate complete delivery of intact NAD^+^ to tissues or organs. Human studies have not established the distribution of the intact NAD^+^ molecule across organs, and rapid extracellular metabolism may contribute substantially to the measured circulating metabolite profile.

The three principal NAD^+^ biosynthesis pathways (*de novo*, Preiss-Handler, and salvage) and the contrasting pharmacokinetic fates of IV *versus* oral precursor delivery are illustrated in Figure 1.

### 3.2 Clinical evidence for NAD^+^ therapy in wellness applications

This section presents clinical evidence organised by route of administration. IV NAD^+^ and IV NMN human studies are presented first, followed by oral NAD^+^ precursor human studies. Preclinical mechanistic evidence relevant to clinical translation is addressed in the Mechanisms of Action section and the Discussion.

#### 3.2.1 IV NAD^+^ and IV NMN: human clinical studies

The most comprehensive human study of IV NAD^+^ therapy to date enrolled eight healthy elderly adults aged 70–80 years in an open-label, uncontrolled pilot design (Braidy, 2018). Participants received 1,000 mg IV NAD^+^ (approximately 1.51 mmol) daily for six consecutive days. Post-infusion assessments demonstrated marked elevation of circulating NAD^+^ metabolome components, alongside significant reductions in oxidative stress biomarkers (8-iso-prostaglandin F2α, advanced oxidative protein products, protein carbonyl content) and pro-inflammatory cytokines (CRP, IL-6), and activation of longevity-associated signalling pathways (increased SIRT1 and FOXO1 expression, decreased acetylated p53). Key limitations include the absence of a control or placebo arm, very small sample size (n = 8), lack of randomisation or blinding, and a follow-up period restricted to the 6-day infusion window; findings must therefore be treated as preliminary and hypothesis-generating rather than confirmatory. Full dosing characteristics, molar equivalents, and safety data are summarised in Tables 1, 2.

**TABLE 1:** Summary of human studies evaluating IV NAD^+^ therapy and oral NAD^+^ precursor supplementation.

Study	Study purpose	Design	Route	Intervention/Dose	n	Nicotinamide (NAM) (Vit B3) equivalency per infusion (mmol NAM)	Main findings	Major limitations
IV NAD^+^ studies								
Braidy (2018)	Safety/Pharmacodynamic assessment	Open-label, uncontrolled pilot	IV NAD^+^	1,000 mg/day × 6 days	8 (70–80 years)	∼1.51 mmol NAM equiv./infusion	Marked elevation of NAD^+^ metabolome; increased SIRT1 activity; reduced oxidative stress and systemic inflammation markers	No control arm; very small n; no randomisation; short duration
Rutherford et al. (2020)	Case report/Neurological outcome	Single-patient case report	IV NAD^+^	500–1500 mg NAD^+^	1 (Parkinson’s disease)	∼0.75–2.26 mmol NAM equiv./infusion	Significant reduction in objective wrist accelerometry-measured tremor severity across infusion course	Single patient; no causal inference; no control; unknown dose and duration
Grant et al. (2019)	PK/metabolomic study	Controlled pilot study	IV NAD^+^	750 mg NAD^+^ over 6 h (∼3 μmol/min)	11 (8 NAD^+^ 3 saline control)	∼1.13 mmol NAM equiv./infusion	Time-dependent increases in plasma NAD^+^, NAM, ADPR, meNAM and NMN; urinary NAD^+^ and meNAM also increased. Early circulating removal was followed by later metabolite accumulation	Small sample; short follow-up; exploratory PK/metabolomic outcomes; no clinical efficacy endpoints
Reyna et al. (2026)	Real-world route comparison (NAD^+^ vs. NR)	Retrospective real-world comparison	IV NAD^+^/IV NR	Variable clinic doses	14 (6 NAD^+^, 8 NR)	Variable	Both IV NAD^+^ and IV NR increased blood NAD^+^; preliminary route-specific metabolomic differences noted; pilot observational data only	Very small n; retrospective; no controls; potential confounders; insufficient power for comparative conclusions
IV NMN studies								
Kimura et al. (2022)	Primary safety assessment	Open-label, exploratory	IV NMN	300 mg IV NMN; not fully specified; multiple infusion sessions	10 (healthy adults)	∼0.90 mmol NAM equiv./infusion	No adverse cardiovascular or metabolic events; preliminary signal for improved sleep quality	Uncontrolled; unblinded; exploratory endpoints; NMN rather than NAD^+^
Oral NAD^+^ precursor studies (comparative context)								
Martens et al. (2021)	Clinical efficacy (metabolic/insulin sensitivity)	RCT, double-blind, placebo-controlled	Oral NMN	250 mg/day × 10 weeks	30 (postmenopausal women with pre-diabetes)	∼0.75 mmol NAM equiv./day	Improved skeletal muscle insulin signalling and gene expression; modest NAD^+^ increases in muscle	Short duration; single sex; no direct NAD^+^ metabolomic profiling in blood
Yoshino et al. (2021)	Clinical efficacy (metabolic)	RCT, double-blind, placebo-controlled	Oral NMN	250 mg/day × 10 weeks	25 (postmenopausal women with overweight/obesity)	∼0.75 mmol NAM equiv./day	Increased muscle NAD^+^-related metabolite concentrations; improved insulin sensitivity in those with baseline impairment	All female; single-site; modest sample size; no blood NAD^+^ data
Pencina et al. (2023)	PK/Pharmacodynamic dose-finding benchmark	RCT, double-blind, placebo-controlled, dose-escalation	Oral β-NMN (MIB-626)	1,000 mg/day or 2,000 mg/day × 28 days	30 (middle-aged/older; cardiometabolic risk)	∼2.99 mmol NAM equiv./day at 1,000 mg; ∼5.98 mmol/day at 2,000 mg	Dose-dependent ∼3× increase in whole-blood NAD^+^ at 2,000 mg/day; no serious AEs; rigorous benchmark for oral NMN pharmacodynamics	Short duration (28 days); predominantly cardiometabolic risk cohort; oral route — no IV comparison
Santangelo et al. (2022)	Open-label clinical efficacy (neurological/cognitive)	Open-label, uncontrolled	Oral NR	1000 mg NR/day for 12 weeks	50 (mild cognitive impairment)	∼3.92 mmol NAM/day	Improvements in cognitive function, mood, and sleep quality reported; preliminary signal for NAD^+^ augmentation in MCI	No control; subjective endpoints; oral route — not directly comparable to IV NAD^+^
Chellappa et al. (2022)	Pharmacokinetics/Gut microbiome interaction	Observational host–microbiome pharmacokinetic study	Oral NMN/Oral NR	Variable precursor doses	Human cohort (variable)	Variable	Bidirectional cycling of NAD^+^ precursors between host tissues and gut microbiome demonstrated; microbiome consumes and modifies oral precursors prior to systemic absorption	Oral route only; microbiome pharmacokinetics not studied for IV route; findings may not translate to IV administration
Systematic reviews and meta-analyses								
Gindri et al. (2024)	Safety and tolerability review (oral precursors)	Systematic review	Oral NMN/NR/NAM	Variable across included trials	Multiple trials aggregated	Variable across included trials	Oral NAD^+^ precursors generally well-tolerated; modest but consistent NAD^+^ increases; acceptable safety profile across dose ranges studied	No IV arm; heterogeneous protocols; short-duration trials dominate

Abbreviations: ADPR, adenosine diphosphoribose; AE, adverse event; NAM, nicotinamide; NaMN, nicotinic acid mononucleotide; PK, pharmacokinetic.

**TABLE 2:** Adverse events and safety data reported across IV NAD^+^ and IV NMN human studies.

Study	Route/Formulation	Dose range	n	Adverse events reported	Serious AEs	Infusion-related reactions	Notes on safety profile
Braidy (2018)	IV NAD^+^	1,000 mg/day × 6 days	8	Mild flushing, transient nausea	None reported	Flushing; transient chest discomfort in some participants	Small uncontrolled study with limited systematic safety assessment; findings should be interpreted cautiously
Grant et al. (2019)	IV NAD^+^	750 mg over 6 h (∼3 μmol/min)	11 (8 NAD^+^; 3 saline control)	No AEs observed during infusion; small changes in selected liver enzymes and bilirubin were reported but were not considered clinically significant	None reported	None observed	Controlled pilot study with short-term follow-up; small sample and limited ability to assess uncommon or delayed adverse events
Kimura et al. (2022)	IV NMN	300 mg	10	No clinically significant adverse events reported	None reported	None observed	Systematic safety assessment was primary endpoint; no cardiovascular or metabolic AEs
Rutherford et al. (2020)	IV NAD^+^	500–1500 mg NAD^+^	1	None reported	None reported	Not systematically described	Single case; limited safety information
Pencina et al. (2023)[oral comparator]	Oral β-NMN (MIB-626)	1,000 mg twice/day × 28 days	30	Mild GI effects at higher dose	None serious; two moderate events	N/A (oral route)	Rigorous AE monitoring; benchmark for oral NMN safety at pharmacodynamic doses

Abbreviations: AE, adverse event.

Kimura et al. (2022) conducted a safety-focused open-label study of IV NMN in ten healthy adult volunteers; specific infusion doses were not fully standardised across sessions and are presented in Table 1 alongside NMN molar equivalents (MW334.2 g/mol). IV NMN increased blood NAD^+^ levels without affecting electrocardiographic parameters, haematological indices, or markers of hepatic, cardiac, pancreatic, or renal function. Participants additionally reported improvements in subjective sleep quality, a signal consistent with the larger oral NMN trial by Kim et al. (2022); n = 108), which demonstrated that time-dependent dosing (ingestion after 18:00 h) — but not morning dosing—produced significant sleep quality improvements, indicating a circadian time-dependent effect. Key limitations include the open-label, uncontrolled design, small sample size (n = 10), use of IV NMN rather than NAD^+^ directly, incompletely specified infusion doses, and the exploratory nature of secondary endpoints such as sleep quality. Full data and molar equivalents are presented in Table 1; adverse event data in Table 2.

Reyna et al. (2026) compared IV NAD^+^ and IV NR in a small real-world observational sample (NAD^+^: n = 6; NR: n = 8) using variable clinic-administered doses; precise doses and corresponding molar equivalents are reported in Table 1. NAD^+^ recipients experienced moderate-to-severe infusion-associated symptoms (nausea, cramps, chest discomfort) with a mean infusion time of 97 min, compared with mild, short-lived effects and a mean infusion time of 37 min for NR. Both agents produced no significant changes in liver enzymes, CRP, or renal function markers over 30 days; alkaline phosphatase declined significantly only in the NAD^+^ group, a between-group difference of uncertain clinical significance. Principal limitations include very small sample sizes in each group, retrospective data collection, absence of a placebo control, potential for substantial confounding from uncontrolled lifestyle variables, and insufficient statistical power for reliable comparative conclusions. Full data are presented in Table 1; safety comparisons in Table 2.

Grant et al. (2019) conducted a controlled pilot pharmacokinetic/metabolomic study in 11 healthy men, with eight participants receiving 750 mg NAD^+^ over 6 h and three receiving saline control. The infusion rate was approximately 3 μmol/min. Plasma NAD^+^, NAM, ADP-ribose, N^1^-methylnicotinamide, and NMN showed time-dependent changes, while urinary NAD^+^ and N^1^-methylnicotinamide excretion also increased. Notably, NAD^+^ and its metabolites showed little change during the early part of the infusion, followed by marked increases later in the 6 h, consistent with rapid initial removal from the circulation and subsequent saturation of metabolic or tissue-handling pathways. The study did not assess clinical efficacy or cognitive outcomes. Its small sample size and short observation period limit interpretation, but the controlled design and detailed sampling make it important for understanding the pharmacokinetic fate of IV NAD^+^ (Grant et al., 2019). Available data are presented in Table 1; safety data in Table 2.

A single case report by Rutherford et al. (2020) described IV NAD^+^ infusion in one patient with Parkinson’s disease. The patient received a specific protocol of NAD^+^ (called BR + NAD) for a total of 6 days, with 2 days of 1,500 mg IV NAD^+^, followed by 4 days of 500–750 mg IV NAD^+^. Treatment was associated with a measurable reduction in wrist accelerometry-assessed tremor severity across the infusion course, an outcome of exploratory interest in the context of NAD^+^-related neuroprotection. Fundamental limitations preclude any generalisable conclusions: a sample size of one means that individual variation cannot be distinguished from treatment effect; the absence of a control condition means that temporal confounders—including regression to the mean and natural symptom fluctuation—cannot be excluded; dose, duration, and administration details are incompletely reported; and no causal inference can be drawn. These findings require confirmation in adequately powered, controlled trials. Available data are presented in Table 1; safety data in Table 2.

#### 3.2.2 Oral NAD^+^ precursor studies: human clinical evidence

Santangelo et al. (2022) conducted an open-label exploratory study of oral NR supplementation (1000 mg/day for 12 weeks) in 50 participants with mild cognitive impairment and mild Alzheimer’s disease. The study reported increased brain NAD^+^ measurements and exploratory cognitive findings. However, this study used oral rather than IV administration, and the open-label design without a placebo control limits the interpretability of findings. These results should therefore not be extrapolated to support IV NAD^+^ therapy in neurodegenerative conditions.

Martens et al. (2021) evaluated oral NMN supplementation in a randomized, double-blind, placebo-controlled trial involving 30 postmenopausal women with prediabetes. Participants received 250 mg NMN daily for 10 weeks. NMN supplementation was associated with improved skeletal muscle insulin signalling and changes in muscle gene expression, along with a modest increase in skeletal muscle NAD^+^ levels. However, the findings should be interpreted cautiously because of the small sample size, short treatment period, inclusion of only women, and lack of direct blood NAD^+^ metabolomic measurements.

Pencina et al. (2023) conducted a randomised, double-blind, placebo-controlled dose-escalation trial of oral MIB-626 (microcrystalline β-NMN) in 30 middle-aged and older adults with cardiometabolic risk factors. At 2,000 mg/day over 28 days, whole-blood NAD^+^ concentration increased approximately 3-fold above baseline without serious adverse events, providing a rigorous pharmacodynamic benchmark against which IV NAD^+^ infusion protocols should be evaluated in future comparative studies (see Table 1).

Henderson et al. (2024) evaluated a multi-component systems approach to NAD^+^ augmentation using oral precursor supplementation and reported exploratory changes in inflammatory and metabolic biomarkers. While mechanistically informative, the oral administration route and heterogeneous outcome measures mean that these findings do not constitute direct evidence for IV NAD^+^ therapy.

#### 3.2.3 Energy metabolism and metabolic health: oral NAD^+^ precursor evidence

As a key cofactor for glycolysis, the tricarboxylic acid cycle, and oxidative phosphorylation, NAD^+^ is central to cellular energy production. Age-related decline in NAD^+^ has been associated with reduced mitochondrial oxidative phosphorylation capacity and impaired cellular energy availability (Janssens et al., 2022). Notably, skeletal muscle NAD^+^ concentrations can be maintained at more youthful levels through regular physical activity, suggesting that interventions to restore NAD^+^ — including IV administration—may be of particular relevance in sedentary or aged populations with impaired NAD^+^ biosynthesis.

Sohouli et al. (2024) and Gindri et al. (2024), in independent systematic reviews, both reported modest and heterogeneous effects of oral NAD^+^ precursor supplementation on glucose metabolism, CRP, liver enzymes, and insulin sensitivity; IV-specific data were limited in both analyses. Two randomised controlled trials by Dollerup et al. (2018), Dollerup et al. (2020) found that high-dose oral NR (2,000 mg/day and 1,000 mg twice-daily, respectively) was safe and well-tolerated in obese, insulin-resistant men but produced no significant improvement in insulin sensitivity, glucose metabolism, or skeletal muscle mitochondrial function—underscoring the recurrent dissociation between NAD^+^ biomarker elevation and clinical metabolic benefit observed across oral precursor trials.

Sharma et al. (2023) proposed that synergistic supplementation of NAD^+^-promoting compounds may represent a strategy for increasing healthspan, noting that NAD^+^ plays a central role in mitochondrial function, inflammatory regulation, and cellular adaptation to stress (Verdin, 2015; Sharma et al., 2023). However, most supporting evidence remains preclinical or derived from oral supplementation studies, and the applicability to IV NAD^+^ therapy specifically remains speculative.

### 3.3 Safety considerations and risk-benefit assessment

The safety evidence for IV NAD^+^ is still limited and mainly comes from small studies, observational reports, and relatively short follow-up periods (Table 2). Although serious adverse events have not been reported consistently, these studies are not sufficient to draw firm conclusions about the long-term safety of IV NAD^+^. The five available human studies were considered in terms of their study design, sample size, randomization, blinding, use of a control group, pre-specified outcomes, and overall risk of bias (Table 3). Taken together, the evidence is weakened by small study populations, short follow-up, and largely exploratory designs, with no adequately powered randomized controlled trials evaluating clinically meaningful outcomes. Grant et al. (2019) was the only study to include a saline control group; however, its small sample and exploratory pharmacokinetic/metabolomic design limit the strength of the conclusions.

**TABLE 3:** Evidence quality and risk-of-bias assessment for IV NAD^+^ and IV NMN human studies.

Study	Study design	Sample size (n)	Randomised	Blinded	Control group	Primary outcome pre-specified	Overall risk of bias
Braidy (2018)	Open-label, uncontrolled pilot	8 (healthy elderly, 70–80 years)	No	No	None	Yes-NAD^+^ metabolome and oxidative stress markers	High
Grant et al. (2019)	Controlled pilot PK/metabolomic study	n = 11 (8 NAD^+^, 3 saline control)	No	No	Yes-saline control	Yes-pharmacokinetic/metabolic outcomes	Some concern
Rutherford et al. (2020)	Single-patient case report	1 (Parkinson’s disease)	No	No	None	No (case report; no pre-specified outcome)	High
Reyna et al. (2026)	Retrospective real-world observational comparison	14 (NAD^+^: n = 6; NR: n = 8)	No	No	None	No (retrospective; no pre-specified primary outcome)	High
Kimura et al. (2022)	Open-label, exploratory safety study	10 (healthy adults)	No	No	None	Yes-safety and tolerability (primary endpoint)	High

Short-term adverse effects reported during IV NAD^+^ administration have included nausea, abdominal discomfort, flushing, cramping, chest pressure, and infusion-related intolerance, particularly at faster infusion rates (Reyna et al., 2026). In comparative observational data, IV NR appeared to be better tolerated than IV NAD^+^ itself, although the studies were small and exploratory (Reyna et al., 2026). Further, NR was well tolerated at the daily oral dose of 2000 mg in a 12-week study in healthy men (Dollerup et al., 2018).

Several important uncertainties remain regarding chronic or repeated NAD^+^ augmentation. Because NAD^+^ participates in multiple metabolic and proliferative pathways, theoretical concerns have been raised regarding potential interactions with malignancy biology and altered cellular proliferation. Although preclinical studies involving NMN did not demonstrate increased tumor burden in animal models (Yoo et al., 2021; Kane et al., 2024-preprint), these findings cannot exclude long-term risk in humans receiving repeated IV administration.

There have also been context-dependent biological effects in preclinical experiments. For example, Qu et al. (2023) reported differing results depending on the timing of NAD^+^ precursor administration in experimental stroke models. That physiologic context, disease state, timing, and dosing likely exert a strong influence on biological responses to NAD^+^ augmentation. Additional uncertainties relate to potential interactions with drugs and various chronic diseases. NAD^+^ modulates various metabolic and signaling pathways; hence, interactions with drug therapies for diabetes, cardiovascular disease, neurologic disorders, and other chronic disorders remain less well characterized. In addition, safety data in patients with severe comorbidities like renal disease, hepatic dysfunction, or active malignancy are very sparse. Regulatory and quality control considerations also need to be considered. Currently, IV NAD^+^ formulations, dosing protocols, and manufacturing consistency across wellness and infusion clinics do not have standardized international guidelines. Variability in preparation methods, infusion practices, and accompanying supplements may affect safety and reproducibility. Hence, IV NAD^+^ treatment must be considered investigational, and more controlled studies with long-term follow-up are still needed to conclude on the safety and risk-benefit profile.

### 3.4 Dose regimens and infusion methods

NAM (vitamin B3) equivalency is a useful metric for comparing doses across studies using different NAD^+^ precursors. Because NAD^+^, NMN, and NR each contain one NAM moiety per molecule, their molar dose corresponds to the molar NAM equivalency on a 1:1 basis. Accordingly, 1,000 mg IV NAD^+^ corresponds to approximately 1.51 mmol NAM equivalents, 1,000 mg NMN to approximately 2.99 mmol, and 1,000 mg NR to approximately 3.92 mmol. In the Grant et al. (2019) study, the administered dose was explicitly reported as 750 mg NAD^+^ over 6 h, corresponding to approximately 1.13 mmol NAM equivalents per infusion. The Braidy (2018) infusion of 1,000 mg NAD^+^/day therefore provided approximately 1.51 mmol NAM equivalents per infusion, whereas the Kimura et al. (2022) study used 300 mg IV NMN, equivalent to approximately 0.90 mmol (Table 1). Reporting both the administered mass and molar NAM equivalency improves comparability across studies and avoids misleading gram-dose comparisons between chemically different compounds.

IV NAD^+^ administration may require logistical considerations such as duration of infusion, a diluent formulation, infusion rate, and monitoring. Outpatient clinic infusions with monitoring have been described in published literature, but specific infusion rate and diluent information are not standardized and vary (Braidy, 2018). Rapid IV NAD^+^ infusion can sometimes induce discomfort, flushing, or nausea according to anecdotal reports from clinical wellness settings where many practitioners prefer slow infusion rates extending from 2 to 4 h. Systematic dose-escalation studies and direct comparisons of different IV NAD^+^ doses remain lacking. The available studies used different protocols and doses, limiting meaningful dose–response comparisons.

## 4 Discussion

This review highlights the considerable gap between the growing commercial adoption of IV NAD^+^ therapy and the limited quality of currently available clinical evidence. Although mechanistic studies provide a biologically plausible rationale for NAD^+^ augmentation, clinical evidence supporting meaningful therapeutic or wellness-related benefits remains preliminary. Existing human studies suffer from small sample sizes, the lack of suitable controls, heterogeneous methodologies, and short-term follow-up, making it difficult to ascertain whether known biomarker alterations translate into clinically meaningful effects. The available literature should be interpreted with caution. Although some reports show changes in inflammatory markers, oxidative stress parameters, or subjective patient-reported outcomes, these results are not definitive in terms of anti-aging, cognitive enhancement, metabolic optimization, or disease prevention. At present, the evidence is insufficient to support routine clinical implementation of IV NAD^+^ therapy outside appropriately designed research settings.

### 4.1 Interpretation of clinical evidence

The study by Braidy (2018), the most methodologically detailed IV NAD^+^ human study identified in this review, provides proof-of-concept for pharmacodynamic activity of repeat-dose IV NAD^+^ in elderly adults (full descriptive data in Tables 1 and 2). The key interpretive point is that although post-infusion biomarker changes were statistically significant and biologically coherent (SIRT1 activation, reduction in oxidative stress and inflammatory markers), the absence of a placebo control arm, the n = 8 sample size, and the lack of randomisation constrain these findings to proof-of-concept status. They demonstrate that IV NAD^+^ achieves meaningful pharmacodynamic activity *in vivo*, but cannot establish therapeutic efficacy or distinguish between pharmacological and non-specific infusion effects.

The IV NMN study by Kimura et al. (2022) extends the short-term safety characterisation for IV NAD^+^ precursors beyond the NAD^+^ molecule itself (data in Tables 1 and 2). Interpretively, the confirmation of acceptable cardiovascular and metabolic safety in ten healthy volunteers is clinically relevant, but the open-label, unblinded, single-arm design precludes any efficacy inference. The preliminary sleep quality signal is hypothesis-generating but requires replication in a placebo-controlled study before it can be attributed to IV NMN pharmacodynamics rather than expectation effects or infusion-related relaxation.

The single-patient Parkinson’s case study by Rutherford et al. (2020) is noteworthy for its use of objective wrist accelerometry to quantify tremor severity, providing quantitative outcome data that contrasts with the subjective endpoints used in most other reports in this space (full details in Tables 1 and 2). Nevertheless, no causal inference can be drawn from a single uncontrolled case, and replication in adequately powered, blinded, controlled trials is required before IV NAD^+^ can be considered a candidate intervention in neurological disease.

Oral NAD^+^ precursor studies provide important pharmacodynamic context for interpreting IV NAD^+^ findings. Trammell et al. (2016) and Elhassan et al. (2019) established that oral NR is bioavailable and elevates circulating and tissue NAD^+^ metabolites in healthy humans, demonstrating that meaningful systemic NAD^+^ augmentation does not require the IV route. The critical interpretive question is therefore not whether IV NAD^+^ achieves higher peak plasma concentrations than oral supplementation; it does, because IV administration bypasses gastrointestinal absorption and first-pass metabolism of the administered dose and provides direct systemic exposure; however, this does not establish complete bioavailability of intact NAD^+^ at tissues or organs, but whether this transient supraphysiological exposure translates into superior or distinct clinical outcomes.

Two placebo-controlled trials by Dollerup et al. (2018), Dollerup et al. (2020) provide a cautionary interpretive frame for the whole field: both trials confirmed dose-dependent NAD^+^ metabolite elevation with high-dose oral NR, yet neither demonstrated improvements in insulin sensitivity, glucose metabolism, or skeletal muscle mitochondrial function in obese, insulin-resistant men. This dissociation-robust pharmacodynamic signal, absent clinical benefit-recurs across oral precursor trials and is directly applicable to IV NAD^+^: demonstrating that an IV infusion elevates whole-blood NAD^+^ does not, in itself, establish that downstream clinical benefits follow. Well-designed, outcome-powered controlled trials using IV NAD^+^ remain the necessary and currently absent evidence base.

### 4.2 Comparison with oral NAD^+^ precursor supplementation

The critical interpretive question is therefore not simply whether IV NAD^+^ produces higher peak circulating concentrations than oral supplementation, but whether this exposure translates into superior or distinct clinical outcomes. IV administration places the administered dose directly into the circulation and can produce rapid plasma exposure, but this should not be equated with complete bioavailability of intact NAD^+^ at the tissue or organ level. The distribution of intact NAD^+^ after IV administration has not been demonstrated in humans, and extracellular metabolism may occur before or alongside tissue uptake.

Potential formation of extracellular purine metabolites, including adenosine, following NAD^+^ breakdown represents a pharmacologically distinct and currently understudied consequence of IV NAD^+^ administration. Activation of A1, A2A, A2B, and A3 purinergic receptors by adenosine produced during extracellular NAD^+^ metabolism may contribute to some infusion-associated sensations and may also contribute to putative vasodilatory and immunomodulatory effects, although these effects have not been established as specific to IV NAD^+^ therapy. This extracellular signalling pathway may be particularly relevant to IV administration because IV delivery produces direct systemic exposure to administered NAD^+^. Still, its contribution to clinical effects remains uncertain and should not be considered exclusive to the IV route.

The NNMT-mediated irreversible loss of NAM to MeNam represents a pharmacokinetic inefficiency that is invisible to conventional whole-blood NAD^+^ measurements, since the metabolic fate of NAM released during NAD^+^ hydrolysis is not captured by standard NAD^+^ assays. Inter-individual variation in hepatic and adipose NNMT activity may therefore contribute substantially to the heterogeneity in whole-blood NAD^+^ responses observed across IV NAD^+^ studies. Urinary MeNam excretion provides a non-invasive proxy for NNMT flux and should be included as a secondary endpoint in future pharmacokinetic trials of IV NAD^+^, to enable more complete mass-balance accounting of administered NAD^+^ and to facilitate cross-study comparisons of metabolic efficiency.

The bidirectional cycling of NAD^+^ precursors between host tissues and the gut microbiome, characterised by Shats et al. (2020) and Chellappa et al. (2022), introduces a source of inter-individual pharmacokinetic variability in oral precursor trials that is entirely circumvented by IV administration. Individuals with divergent gut microbial compositions may therefore exhibit markedly different plasma NAD^+^ responses to identical oral NMN or NR doses, potentially contributing to the inconsistent efficacy signals observed across oral precursor RCTs. Whether IV NAD^+^ provides greater bioavailability of intact NAD^+^ at the tissue or organ level than oral supplementation remains untested in direct comparative human pharmacokinetic studies, and this represents an important priority for future research.

Extracellular NAD^+^ metabolism may also represent a pharmacological difference between IV NAD^+^ and oral precursor administration. NAD^+^ can be cleaved by extracellular glycohydrolases such as CD38, while other extracellular nucleotide-processing activities can generate NMN, AMP, and subsequently adenosine. CD73 is well established as an AMP phosphatase that produces adenosine, but the extent to which human CD73 itself contributes directly to NAD^+^ or NMN processing remains uncertain. Wilk et al. (2020) specifically found little or no direct processing of NAD^+^ by recombinant human CD73. These distinctions are important because they prevent the entire extracellular NAD^+^ catabolic sequence from being assigned to a single enzyme.

The cost implications also support oral supplementation for most wellness applications. IV administration involves clinical infrastructure, trained personnel, monitoring equipment, and time commitments that significantly increase costs relative to oral supplements. Whether the clinical benefits of IV administration are worth these additional expenses and commitments is left to be seen. Ritual and experience in IV therapy could add psychosocial benefits beyond pharmacological benefits in the case of such a patient, yet a solid basis for such an effect cannot be found.

### 4.3 Mechanisms and biological plausibility

A further issue is the interpretation of blood NAD^+^ measurements themselves. Circulating NAD^+^ is not a static biomarker: measured values can be influenced by the timing of collection, fasting status, sample processing, storage temperature, and freeze–thaw history. Recent multi-cohort work showed that pre-analytical and analytical variation can materially affect measured whole-blood NAD^+^ concentrations and may obscure or create apparent biological differences (Trętowicz et al., 2026). This is particularly relevant to IV studies, where short-lived changes may be interpreted as evidence of systemic NAD^+^ repletion. Future trials should therefore standardize the time of blood collection relative to infusion and meals, process samples promptly, use validated NAD^+^ preservation procedures, and report storage and analytical conditions in detail.

### 4.4 Preclinical mechanistic evidence: translational context

Much of the mechanistic rationale supporting NAD^+^ augmentation derives from preclinical studies. Experimental models have demonstrated associations between increased NAD^+^ availability and activation of pathways related to mitochondrial function, oxidative stress regulation, inflammatory signaling, and cellular stress responses (Verdin, 2015). However, these mechanistic observations should not be interpreted as confirmation of clinical efficacy in humans.

Several studies have suggested that NAD^+^-dependent activation of sirtuin pathways, particularly SIRT1, may influence cellular adaptation to metabolic stress (Verdin, 2015). In a small exploratory human study, IV NAD^+^ administration was associated with increased SIRT1 activity and altered expression of selected downstream signaling markers (Braidy, 2018). Although these findings suggest biologic activity, their clinical significance remains uncertain.

Similarly, experimental evidence indicates that NAD^+^ supplementation may influence oxidative stress and inflammatory pathways. Zhang et al. (2018) described mechanistic interactions involving SIRT2-, ERK-, and Nrf2-related signaling pathways in preclinical models. While reductions in oxidative stress markers have been observed following NAD^+^ administration, these biomarker changes do not establish clinically meaningful anti-aging or disease-modifying effects.

Preclinical studies evaluating NMN supplementation in mice have reported effects on insulin sensitivity, mitochondrial activity, frailty progression, and lifespan-related outcomes (Kane et al., 2024-preprint). Sex-specific responses were also observed in some animal models. However, these findings remain exploratory and may not translate directly to human physiology.

Neuroprotective observations in preclinical models, including prevention of chemotherapy-associated cognitive changes, further support biologic plausibility for NAD^+^ augmentation (Yoo et al., 2021). Nevertheless, important uncertainties remain regarding blood–brain barrier transport, tissue compartmentalization, and the ability of systemic IV administration to produce clinically meaningful central nervous system effects.

Overall, the mechanistic literature provides biologic rationale for continued investigation of NAD^+^ augmentation strategies. However, substantial gaps remain between mechanistic plausibility and demonstration of validated clinical benefit in humans.

## 5 Limitations of the review

Several limitations should be considered when interpreting this review. First, this manuscript was conducted as a structured narrative review rather than a formal systematic review or meta-analysis due to the limited number of controlled clinical studies and the marked heterogeneity in the available literature. Consequently, study selection and interpretation remain inherently more subjective.

Second, a structured evidence quality and risk-of-bias assessment of the identified IV human studies is provided in Table 3. The available evidence is predominantly based on small exploratory studies, and most studies did not use randomisation or blinding. Grant et al. (2019), however, included a small saline control group, so the IV evidence should not be described as uniformly uncontrolled.

Third, the existing literature presents significant heterogeneity in patient populations, infusion protocols, NAD^+^ formulations, outcome measures, and duration of follow-up. Such variations do not allow direct comparison between studies, leading to limited opportunities for meaningful quantitative synthesis.

Fourth, multiple studies cited utilize oral NAD^+^ precursors rather than IV NAD^+^ administration itself. Although these studies generate critical mechanistic and translational context, they should not be considered as direct evidence in support of IV NAD^+^ therapy.

Finally, the pharmacokinetic fate of intravenously administered NAD^+^ in humans is poorly characterized, especially regarding whether intact NAD^+^ reaches intracellular compartments or is extensively metabolized extracellularly. Circulating blood NAD^+^ levels should therefore not be taken as a direct indication of NAD^+^ levels within specific organs or tissues. In addition, measured blood NAD^+^ concentrations can be affected by several factors, including the timing of blood collection, fasting status, sample storage conditions, and freeze–thaw handling. Such differences may partly explain the variability between studies and should be carefully controlled and reported in future pharmacokinetic studies.

Although every effort was made to prioritize peer-reviewed evidence, one cited study (Kane et al., 2024-preprint) was available only as a preprint at the time of submission. Consequently, its findings should be considered preliminary and interpreted cautiously until independent peer review has been completed.

## 6 Future perspectives

IV NAD^+^ in wellness has reached a critical stage where commercial enthusiasm and clinical use have been faster than the supporting scientific evidence. For this reason, the priority is to have large, well-designed RCTs to ascertain if IV NAD^+^ is clinically useful. These studies must include an array of populations, involve validated biomarkers and patient-centered outcomes, apply rigorous blinding and placebo controls, and include sufficient follow-up to determine durability and safety. Comparing IV *versus* oral NAD^+^ precursors is also important in establishing whether IV therapy provides substantial benefits that support its increased cost and complexity. Concurrently, dose-response studies, pharmacokinetic tests, and long-term safety studies are needed to establish the best protocols and to assess sustained safety and efficacy.

Apart from efficacy trials, the field will also benefit from mechanistic studies in human tissues, biomarker validation, and individualized strategies that establish which individuals are likely to respond. Comparative effectiveness and cost-effective studies and combinations of interventions might help position IV NAD^+^ in the context of general healthcare interventions. Clinician training, clearer regulatory standards, and transparent patient education are equally essential to safe and ethical implementation. And while the biological rationale for the augmentation of NAD^+^ is compelling, the future path forward for IV NAD^+^ therapy is grounded in ongoing, transparent research efforts that can translate initial promise into reliable, evidence-based practice.

## 7 Conclusions

Current evidence relating to IV NAD^+^ therapy remains preliminary, heterogeneous, and methodologically limited. Small exploratory studies have demonstrated transient increases in circulating NAD^+^ levels, along with changes in selected biomarkers associated with oxidative stress, inflammation, and cellular metabolism. However, the clinical significance of these findings remains uncertain, and evidence supporting claims of anti-aging, cognitive enhancement, metabolic optimization, or improved wellness is currently insufficient.

Although mechanistic and translational studies provide a biological rationale for NAD^+^ augmentation, substantial gaps remain between experimental findings and validated clinical outcomes in humans. Major unanswered questions involve optimal dosing strategies, comparative effectiveness *versus* oral supplementation, long-term safety, durability of response, and identification of clinically meaningful endpoints.

At present, IV NAD^+^ therapy should be considered investigational, and its widespread use in wellness settings remains ahead of the available clinical evidence. Future progress in this field will require adequately powered RCTs, standardized treatment protocols, rigorous safety monitoring, and clinically relevant outcome measures before routine clinical implementation can be justified.
